# Clinical predictors of survival in patients with relapsed/refractory small-cell lung cancer treated with checkpoint inhibitors: a German multicentric real-world analysis

**DOI:** 10.1177/17588359221097191

**Published:** 2022-06-04

**Authors:** Jan A. Stratmann, Radha Timalsina, Akin Atmaca, Vivian Rosery, Nikolaj Frost, Jürgen Alt, Cornelius F. Waller, Niels Reinmuth, Gernot Rohde, Felix C. Saalfeld, Aaron Becker von Rose, Fabian Acker, Lukas Aspacher, Miriam Möller, Martin Sebastian

**Affiliations:** Department of Internal Medicine, Hematology/Oncology, Goethe University, Theodor Stern Kai 7, 60596 Frankfurt am Main, Germany; Department of Internal Medicine, Hematology/Oncology, Goethe University, Frankfurt, Germany; Department of Oncology and Hematology, Krankenhaus Nordwest, UCT-University Cancer Center, Frankfurt, Germany; Department of Medical Oncology, West German Cancer Center, University Medicine Essen, Essen, Germany; Charité – Universitätsmedizin Berlin, Berlin, GermanyHumboldt-Universität zu Berlin, Berlin, Germany; Department of Infectious Diseases and Pulmonary Medicine, Berlin Institute of Health, Berlin, Germany; Department of Internal Medicine III (Hematology, Oncology, Pneumology), University Medical Center Mainz, Mainz, Germany; Internal Medicine I, Haematology, Oncology and Stem Cell Transplantation, Faculty of Medicine, Freiburg University Medical Center, Freiburg, Germany; Department of Oncology, Asklepios Clinic München-Gauting, Gauting, Germany; Department of Respiratory Medicine, Medical Clinic 1, University Hospital, Frankfurt, Germany; Department for Internal Medicine I, University Hospital Carl Gustav Carus Dresden, TU Dresden, Dresden, Germany; Department of Internal Medicine III, Klinikum rechts der Isar, Technical University Munich, Munich, Germany; Department of Internal Medicine, Hematology/Oncology, Goethe University, Frankfurt, Germany; Department of Internal Medicine, Hematology/Oncology, Goethe University, Frankfurt, Germany; Department of Oncology, Martha–Maria Hospital Halle, Halle, Germany; Department of Internal Medicine, Hematology/Oncology, Goethe University, Frankfurt, Germany

**Keywords:** checkpoint inhibitor, immunotherapy, metastasis, prognostic biomarker, small-cell lung cancer

## Abstract

**Objectives:**

Small-cell lung cancer (SCLC) is a lung malignancy with high relapse rates and poor survival outcomes. Treatment-resistant disease relapse occurs frequently and effective salvage therapies are urgently needed.

**Materials and Methods:**

We aimed to define efficacy and safety of checkpoint inhibitors (CPIs) in a heterogeneous population of relapsed and refractory SCLC patients in a large retrospective multicentric real-world cohort across German tertiary care centers.

**Results:**

A total of 111 patients from 11 treatment centers were included. Median age of all patients was 64 years, and 63% were male. Approximately one-third of all patients had poor performance status [Eastern Cooperative Oncology Group (ECOG) ⩾ 2], and 37% had known brain metastases. Patients were heavily pretreated with a median number of prior therapy lines of 2 (range, 1–8). Median follow-up of the entire cohort was 21.7 months. Nivolumab and Nivolumab/Ipilimumab were the most common regimens. Overall disease control rate was 27.2% in all patients and was numerically higher in CPI combination regimens compared with single-agent CPI (31.8% *versus* 23.8%; *p* = 0.16). Median overall survival (OS) was 5.8 months [95% confidence interval (CI), 1.7–9.9 months]. The 12- and 24-month survival rates were 31.8% and 12.7%, respectively. The 12-week death rate was 27.9%. Disease control and response rate were significantly lower in patients with liver metastases. Platinum sensitivity (to first-line treatment), metastatic burden, and lactate dehydrogenase (LDH) showed prognostic impact on survival in univariate analysis. Neutrophil-to-lymphocyte ratio (NLR) was a significant and independent predictor of survival in univariate (*p* = 0.01) and multivariate analyses [hazard ratio (HR), 2.1; 95% CI = 1.1–4.1; *p* = 0.03].

**Conclusion:**

CPI in patients with relapsed or refractory (R/R) SCLC is of limited value in an overall patient cohort; however, long-term survival, in particular with CPI combination strategies, is possible. Clinical characteristics allow a more differentiated subgroup selection, in particular patients with low NLR showed less benefit from CPI in R/R SCLC.

## Introduction

Small-cell lung cancer (SCLC) is a lung malignancy that originates from neuroendocrine cells located in the bronchial tree. Due to its aggressive nature in the sense of short tumor doubling time and early metastatic spread, approximately 70% of all patients already have detectable distant metastasis at first diagnosis and up to 23% develop brain metastasis during their course of disease.^1^ Despite high response rates to platinum doublet chemotherapy, acquired treatment resistance frequently occurs within months and the prognosis remains poor with 5-year survival rates below 5% for extensive disease patients, thus underscoring the medical need for effective salvage strategies.^1^ In addition, about one in three SCLC patients has a poor performance status [Eastern Cooperative Oncology Group (ECOG) ⩾ 2], which is associated with even inferior survival times.^2,3^

For more than a decade, no new substances were approved to treat relapsed or refractory (R/R) advanced or metastatic disease despite a multitude of prospective clinical trials, including cytotoxic agents,^4–10^ antibodies,^11,12^ and targeted therapies.^13–17^

With the introduction of checkpoint inhibitors (CPIs), several prospective clinical trials have evaluated its efficacy and safety in patients with R/R SCLC.^18–24^ Based on data of the Checkmate032, Keynote158, and Keynote028 trial, Nivolumab and Pembrolizumab were both temporarily granted approval for the treatment of R/R SCLC in third line or beyond by the Food and Drug Administration (FDA; Nivolumab, 08/2018–01/2021; Pembrolizumab 10/2017–03/2021). Response rates were moderate; however, a small proportion of patients obtains sustainable clinical benefit and long-term responses have been reported.^18^ Of note, patients with poor performance status were excluded from prospective clinical trials; therefore, efficacy and safety of CPI in this considerable proportion of patients is widely unknown.

Several clinical and disease characteristics, such as tumor mutation burden (TMB), PD-L1 expression, tumor-infiltrating immune cells, or even neurological immune-related adverse events have been proposed as predictive biomarkers for disease response upon CPI treatment, but such concepts still lack robust evidence for guiding proper patient selection.^25^

In view of the scant evidence, we performed a multicenter retrospective analysis to shed more light into the field of CPI in patients with R/R SCLC in a real-world population. We aimed to further define populations at risk for inferior outcomes upon CPI treatment and focused on patients with low performance status and brain metastases who were underrepresented in prospective trials.

## Material and methods

We retrospectively analyzed SCLC patients treated within an informal network of 13 cancer centers across Germany, of which 11 centers were able to contribute patient data. Cases were included if they met all of the following criteria: R/R SCLC, CPI treatment – either single agent or CPI combination use – after at least one non-curative treatment line; all patients who had received CPI within a clinical trial or were planned but did not receive CPI treatment were excluded. Clinical information was retrospectively collected from the medical charts.

In Germany, the use of CPI in the context of R/R SCLC has not been approved by the European Medical Agency, but due to the limited treatment options available and in particular in light of the poor prognosis, reimbursement from the health insurance can be applied for as an individual therapeutic trial.

Tumor response was evaluated according to the principles set forth by RECIST 1.1 by the individual treatment centers. Central review was not performed. The rate of non-progression was termed the disease control rate (DCR), and tumor response rate (RR) was defined as the sum of complete response (CR) and partial response (PR).

Time-point endpoints included progression-free survival (PFS) and overall survival (OS). Patients without target events were censored at last follow-up. Adverse events were reported qualitatively with focus on immune-related adverse events (irAEs). Permanent treatment discontinuation due to adverse events was documented.

The number of all included patients and recorded variables were reported using descriptive statistics. Between-group differences were evaluated using a Mann–Whitney or *t* test for continuous data and the chi-square test or Fisher’s exact test for categorical data. Survival analyses were performed using the Kaplan–Meier method for estimation of the percentage of surviving patients, and the log-rank test was used for comparing patient groups. Cox regression was used for multivariate survival analyses. Follow-up was calculated using the reverse Kaplan–Meier method suggested by Schemper and Smith.^26^ A *p*-value below 0.05 was considered statistically significant.

## Results

### Patient characteristics and treatment

Altogether 111 patients were treated with CPI in 11 tertiary treatment centers in Germany between January 2017 and April 2021 (data cut-off). Clinical characteristics are summarized in Table 1 and correspond to the status before start of CPI therapy. Median age of all patients was 64 years, and 63% were male. Almost all patients were active or former smokers. Approximately one-third of all patients had poor performance status (ECOG ⩾ 2), and 37% had known brain metastases. There was no evidence for a predominance of patients with low tumor burden, since 70% of the population had more than 5 metastases, 40.5% of all patients had 3 or more metastatic sites (e.g. lung, bone, liver, and brain). Patients were heavily pretreated with a median number of prior therapy lines of 2 (range, 1–8). All patients had received a platinum-based first-line therapy [cisplatin-based, *n* = 52 (46.8%), carboplatin-based, *n* = 59 (53.1%)]. Median first-line PFS was 7.9 months [95% confidence interval (CI) = 6.7–9.0 months]. The most common regimens in the second-line setting (*n* = 75, 67.6%) consisted of topotecan (*n* = 32, 28.8%) and anthracycline-based therapies (*n* = 28, 25.2%), and third-line therapies were mostly based on previously mentioned treatments, platinum–rechallenge, and the application of paclitaxel-containing strategies. 16.0% (*n* = 18) of all patients had received more than three previous therapy lines (Table 2).

**Table 1.** Clinical characteristics.

		All patients, *n* = 111	
Age in years	Median (range)	63.6 (40.6–80.0)	
Gender	Female	41	36.9%
Smoking history	Never smoker	5	4.5%
	Active smoker	43	38.7%
	Ex smoker	54	48.6%
	Unknown	9	8.1%
ECOG performance status	0	19	17.1%
	1	51	45.9%
	2	22	19.8%
	3	7	6.3%
	Unknown	12	10.8%
Number of previous therapy lines	Median (range)	2 (1–8)	
	1 previous line	29	25.7%
	2 previous lines	46	40.7%
	3 previous lines	18	15.9%
	4 previous lines	15	13.3%
	5 previous lines	2	1.8%
	8 previous lines	1	0.9%
Liver metastases	No	61	55.0%
	Present	47	42.3%
	Unknown	3	2.7%
Brain metastases	No	68	61.3%
	Present	40	36.0%
	Unknown	3	2.7%
Meningeosis carcinomatosa	No	87	78.4%
	Present	4	3.6%
	Unknown	20	18.0%
Metastases count	Limited disease/local progression only	8	7.2%
	1–2 mets	16	14.4%
	3–5 mets	8	7.2%
	>5 mets	75	67.6%
	Unknown	4	3.6%
Number of involved metastatic sites/organs	None (local progression only)	8	7.5%
	1 system	25	22.5%
	2 systems	33	29.7%
	3 systems	32	28.8%
	4 or more systems	13	11.7%
Response to platinum first-line treatment	Sensitive	25	22.5%
	Resistant	55	49.5%
	Unknown	31	27.9%
Serum sodium (mmol/L)	Median (range)	139 (123–147)	
	Hyponatremic	14	14.6%
Serum LDH (U/L)	Median (range)	297 (113–7682)	
Blood lymphocytes (×10E9/L)	Median (range)	0.82 (0.09–2.76)	
Blood neutrophils (×10E9/L)	Median (range)	4.85 (1.04–27.19)	

ECOG, Eastern Cooperative Oncology Group; LDH, lactate dehydrogenase.

Clinical and disease characteristics correspond to the status before checkpoint inhibitor treatment.

**Table 2.** Treatment strategy and disease control rate according to CPI treatment strategy.

Nivolumab 3 mg/kg Q2W							58	51.8%
Atezolizumab 1200 mg Q3W							1	0.9%
Nivolumab 240 mg Q2W							5	4.5%
Nivolumab 1 mg/kg, Ipilimumab 3 mg/kg^a^							45	40.2%
Nivolumab 3 mg/kg, Ipilimumab 1 mg/kg^a^							2	1.8%
		All patients, *n* = 111		Single-agent CPI, *n* = 64		CPI combination, *n* = 47		*p* value
Best response	CR	3	2.9%	2	3.4%	1	2.3%	0.215
	PR	15	14.6%	8	13.6%	7	15.9%	
	SD	10	9.7%	4	6.8%	6	13.6%	
	PD	61	59.2%	40	67.8%	21	47.7%	
Death before radiographic evaluation		14	13.6%	5	8.5%	9	20.5%	
Disease control rate		28	31.5%	14	25.9%	14	40.0%	0.163
Treatment beyond progression	No	78	70.3%	43	67.2%	35	74.5%	0.345
	Yes	33	29.7%	21	32.8%	12	25.5%	

CPI, checkpoint inhibitor; CR, complete remission; PD, progressive disease; PR, partial remission; Q2W, every 2 weeks; Q3W, every 3 weeks; SD, stable disease.

a Q3W for 4 induction cycles, followed by nivolumab single-agent maintenance every 2 weeks.

Nivolumab at a dose of 3 mg/kg bodyweight Q2W was the most often used CPI treatment [*n* = 58 (51.8%)] followed by Nivolumab 1 mg/kg + Ipilimumab 3 mg/kg Q3W for four induction cycles and subsequent Nivolumab maintenance Q2W [*n* = 45 (40.2%)]. The remaining CPI regimens are summarized in Table 2.

Median follow-up of the entire cohort was 21.7 months (95% CI, 9.5–34.0 months).

### Response rates

**Figure 1.**
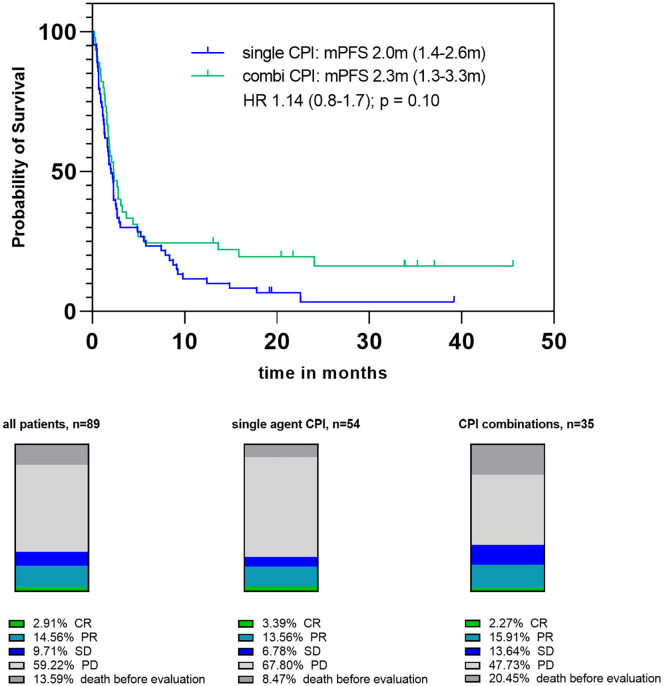
Progression-free survival Kaplan–Meier curves (upper part) and response rates according to checkpoint-inhibitor strategy (combination and single-agent strategy) (lower part).

Data on tumor response were available for 89 patients (80.2%), see Figure 1 and Table 2. Fourteen patients (13.6%) died before radiographic disease evaluation was performed; data were missing for eight patients (7.2%). Overall DCR was 27.2% in all patients, and the overall RR was 17.5%. Median duration of response was 9.8 months [95% CI, 0.0–27.5 months].

DCRs and RR did not differ between patients with good (ECOG 0, 1) *versus* poor (ECOG ⩾2) performance status, presence or absence of brain or bone metastases, male or female sex, single agent or combination treatment strategies, age below or above 65 years, platinum-sensitive or resistant tumors, or neutrophil-to-lymphocyte ratio (<*versus* ⩾ median). DCR (13.9% *versus* 43.4%; *p* = 0.003) and RR (1.9% *versus* 15.5%, *p* = 0.003) were significantly lower in patients with liver metastases. Overall response rate (7.3% *versus* 24.2%, *p* = 0.02), but not DCR (21.9% *versus* 36.8%, *p* = 0.14), was significantly different between patients with more than two metastatic sites compared to two or less. DCR was numerically higher in CPI combination regimens compared with single-agent CPI (DCR, 31.8% *versus* 23.8%; *p* = 0.16) (Figure 1).

### Survival outcomes and risk factors

Median PFS of the entire study cohort was 2.2 months (95% CI, 1.8–2.6 months), and median OS was 5.8 months (95% CI, 1.7–9.9 months). The 12- and 24-month survival rates were 31.8% and 12.7%, respectively.

PFS was not significantly different between patients who were treated with single-agent CPIs compared with combination CPI treatment [2.0 months (95% CI, 1.4–2.6 months) *versus* 2.3 months (95 CI, 1.3–3.3 months); hazard ratio (HR) = 1.1 (95% CI, 0.8–1.7); *p* = 0.10]; however, PFS plateaued at approximately 16% survival, whereas no relevant plateau was seen in patients treated with single-agent CPI (Figure 1).

**Figure 2.**
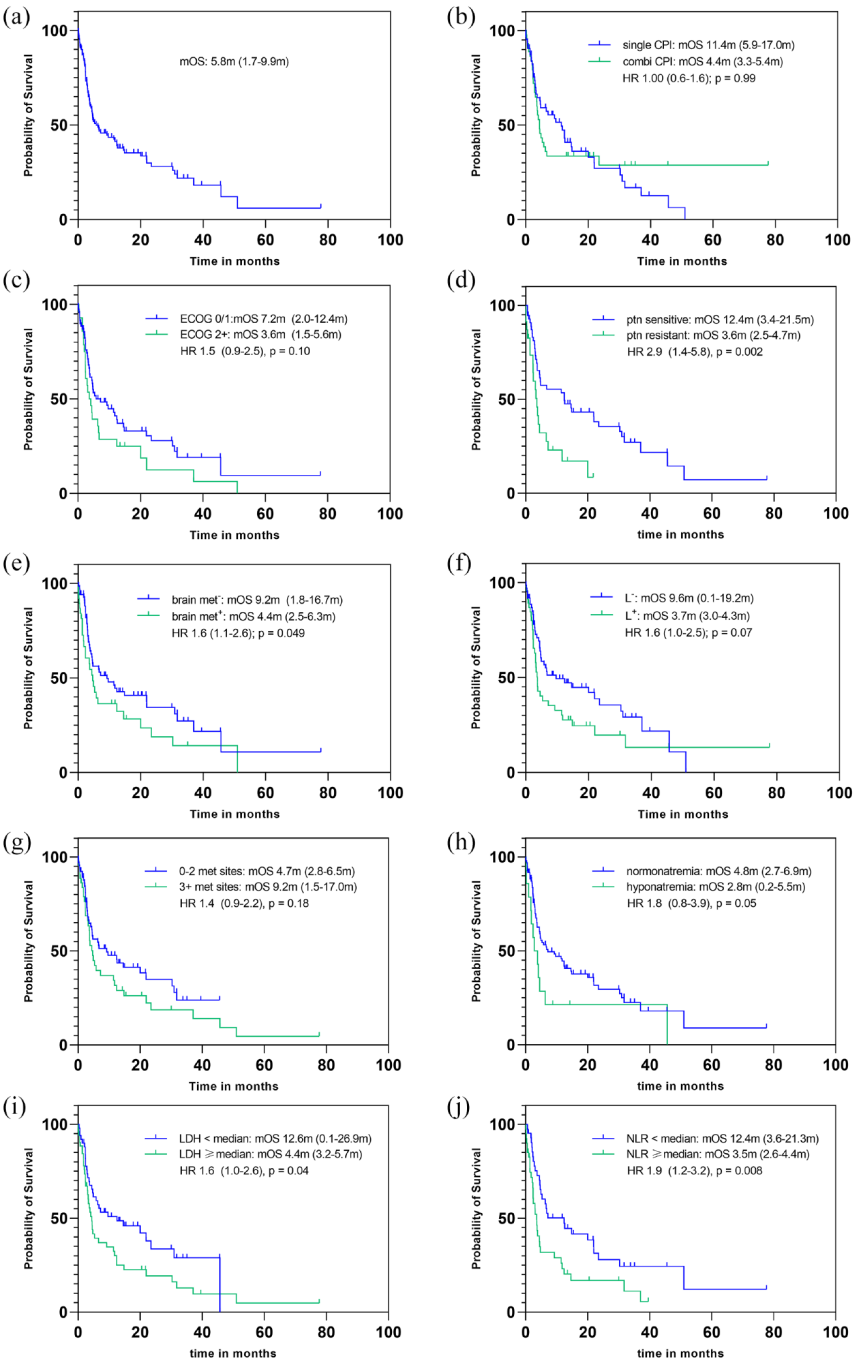
Overall survival Kaplan–Meier curves of the whole cohort (a), according to checkpoint-inhibitor strategy (single agent and combination strategy) (b), according to ECOG performance status (ECOG 0/1 and ECOG performance status) (c), according to platinum sensitivity (d), according to the presence of brain metastasis (e), according to the presence of liver metastasis (f), according to the number of metastatic sites (0–2 metastatic sites and 3+ metastatic sites) (g), according to blood sodium [normonatremia *versus* hyponatremia (cut-off <135 mmol/L)] (h), according to LDH level [<*versus* ⩾ median (297 U/L)] (i), and according to NLR [<*versus* ⩾ median (ratio 5.8)] (j).

There was trend for inferior OS in patients with liver metastases [3.7 months (95% CI, 3.0–4.3 m) *versus* 9.6 months (95% CI = 0.1–19.2 months); HR = 1.6 (95% CI, 1.0–2.5); *p* = 0.07] (Figure 2 and Table 3).

**Table 3.** Risk factors for survival in univariate and multivariate analyses.

	Univariate analysis			Multivariate regression (*n *= 61)		
	HR	95% CI	*p* value	HR	95% CI	*p* value
Age (below or above 65 years)	1.5	0.9–2.5	0.1			
Gender (male *versus* female)	1.1	0.7–1.8	0.6			
ECOG (0–1 *versus* 2+)	1.5	0.9–2.5	0.08			
Platinum sensitivity (sensitivity *versus* resistance)	2.4	1.3–4.3	0.005	1.8	0.9–3.6	0.08
Number of metastatic sites (0–2 *versus* 3+)	1.4	0.9–2.2	0.18			
Metastatic count (0–3 *versus* 4+ metastasis)	2.2	1.2–4.0	0.02	1.2	0.7–1.9	0.35
Presence of brain mets	1.6	1.1–2.4	0.049	1.7	0.9–3.2	0.09
Presence of liver mets	1.5	0.9–2.4	0.07			
Line of therapy (0–2 previous lines *versus* 3+)	0.8	0.5–1.3	0.5			
Type of treatment (single *versus* combination CPI)	1	0.6–1.6	0.2			
Normonatremia *versus* hyponatremia	1.5	0.8–3.0	0.2			
LDH (<*versus* ⩾ median)	1.6	1.0–2.6	0.04	0.8	0.5–2.0	0.8
NLR (<*versus* ⩾ median)	2	1.2–3.3	0.01	2.1	1.1–4.1	0.03
Any irAEs	1.5	0.8–3.1	0.2			

CI, confidence interval; CPI, checkpoint inhibitor; ECOG, Eastern Cooperative Oncology Group; HR, hazard ratio; irAE, immune-related adverse events; LDH, lactate dehydrogenase; NLR, neutrophil-to-lymphocyte ratio.

Presence of brain metastasis [4.4 months (95% CI, 2.5–6.3 months) *versus* 9.2 months (95% CI, 1.8–16.7 months); HR = 1.6 (95% CI, 1.1–2.6); *p* = 0.049], sensitivity to platinum-based first-line therapy [3.6 months (95% CI, 2.5–4.7 months) *versus* 12.4 months (95% CI, 3.4–21.5 months); HR = 2.9 (95% CI, 1.4–5.8); *p* = 0.002], higher (⩾4) count of metastases [4.5 months (95% CI, 3.2–5.8 months) *versus* 30.3 months (95% CI, 0.0–64.0 months); HR = 1.4 (95% CI, 1.2–4.0); *p* = 0.02], in addition to a serum lactate dehydrogenase (LDH) above the median [4.4 months (95% CI, 3.2–5.7 months) *versus* 12.6 months (95% CI, 0.1–26.9 months); HR = 1.6 (95% CI, 1.0–2.6); *p* = 0.04], and an NLR above the median [3.5 months (95% CI, 2.6–4.4 months) *versus* 12.4 months (95% CI, 3.6–21.3 months); HR = 1.9 (95% CI, 1.2–3.2); *p* = 0.0008] was significantly associated with inferior survival (Figure 2 and Table 3).

There was no significant survival difference between patients receiving single agent or combination CPI [HR = 1.0 (95% CI, 0.6–1.6); *p* = 0.99].

In a multivariate regression analysis including the variables platinum sensitivity, overall number of metastases (metastatic count), presence of brain metastases, NLR and LDH, and NLR [< *versus* ⩾ median; HR = 2.1 (95% CI, 1.1–4.1); *p* = 0.03] was the only statistically significant independent adverse risk factors for survival (Table 3).

Thirty-one patients (27.9%) died within 12 weeks after the start of CPI. Of 10 evaluable patients with radiographical evaluation, half (*n* = 5) showed rapid disease progression [+50% sum of longest diameter (SLD) according to RECIST 1.1].

We furthermore compared patients with rapid disease progression (SLD >50%) with those who showed long-term PFS, defined as a PFS >12 months. Supplement S1 shows patient and disease characteristics of rapid progressors and long-term responders in detail. Between group analysis showed significant differences with regard to NLR (< *versus* ⩾ median; *p* = 0.02) and number of metastatic sites (0–2 *versus* 3+; *p* = 0.04) among all variables listed in Table 3. In addition, Supplemental S2 depicts detailed information on patients who died within 12 weeks after CPI initiation.

### Subsequent treatment

Approximately one-fourth of all patients (*n* = 29; 26.1%) received a subsequent treatment line. Treatment strategies were anthracycline-based (*n* = 8), topotecan-based (*n* = 9), or platinum-based (*n* = 7); other therapies were used in five patients. DCR in subsequent lines was poor with 17.2% (*n* = 5). Subsequent survival was not calculated as 42.1% of all events were censored.

### Safety

Table 4 gives an overview on treatment-related adverse events. Combination CPI strategies were associated with a numerically increased toxicity, in particular skin, liver, and endocrinological immune-related adverse events. Treatment discontinuation was non-significantly higher in the CPI combination group. Patients with low performance status tended to have a higher withdrawal rate for non-disease-progression reasons (17.2% *versus* 5.7%; *p* = 0.06), albeit adverse event rate did not differ significantly between patients with poor or good performance status (Supplement S3).

**Table 4.** Immune-related adverse events of all grades.

	All patients, *n *= 63		Single-agent CPI, *n *= 20		CPI combination, *n *= 43		*p* value
irAE: skin toxicity	20	18.0%	3	4.7%	17	36.2%	0.051
irAE: gastrointestinal toxicity	15	13.5%	3	4.7%	12	25.5%	0.26
irAE: liver/pancreas toxicity	7	6.3%	0	0.0%	7	14.9%	0.056
irAE: endocrine toxicity	14	12.6%	1	1.6%	13	27.7%	0.025
irAE: lung toxicity	23	20.7%	6	9.4%	17	36.2%	0.46
irAE: neurological toxicity	6	5.4%	2	3.1%	4	8.5%	0.93
irAE: other	17	15.3%	5	7.8%	12	25.5%	0.8
Permanent discontinuation due to adverse events	10	15.9%	7	6.3%	6	14.0%	0.24

CPI, checkpoint inhibitor; irAE, immune-related adverse events.

## Discussion

We performed a retrospective multicentric analysis of CPI use in R/R SCLC in tertiary care centers across Germany with the aim of outlining its effectiveness and safety in a real-world population with focus on subgroups underrepresented in prospective clinical trials.

Overall, efficacy was moderate with an RR of 17.5% and a median OS of less than 5.8 months which compares well with previously published data from Checkmate032^18,27^ (RR, 11.6–21.9%; OS, 4.7–5.7 months) and pooled data from Keynote028 and Keynote158^21^ (RR, 19.3%; OS, 7.7 months). Checkpoint-inhibitor combination strategies in our cohort revealed a numerically higher response rate that did not translate into a statistically significant survival benefit and therefore mirrored the results from Checkmate032. Nonetheless, indications of a plateau in survival were only seen in the combination regimens and longer follow-up of prospective trials and our cohort is needed for final validation.

Combination CPI treatment was associated with a marked increase of adverse events, in particular skin, liver, and endocrine toxicity; however, permanent discontinuation did not differ between treatment groups in relevant numbers. Conclusively, the choice of a combination strategy over single-agent CPI at the expense of additional side effects including increased treatment costs in the absence of a significant survival benefit in this patient population is currently not recommended outside clinical trials.

Approximately one-third of our cohort had a poor performance status of ECOG 2 or 3, a considerable subgroup of patients that were rigorously excluded from prospective trials in R/R SCLC treated with CPI. It has been well recognized that performance status is an independent predictor of poor outcome among patients treated with chemotherapy in SCLC.^2,28–30^ There was a trend for inferior survival in patients with low performance status in our cohort; nonetheless, response rates and treatment withdrawal due to adverse events did not differ in significant matters between patients with good and poor performance status, thus indicating that CPI treatment was not able to beneficially impact the course of the disease in a sustainable way. A limitation of our study is missing information regarding comorbid conditions and cause-specific death assessment. Although cause of death other than tumor progression may significantly contribute to the overall mortality in very limited stage SCLC,^31^ the aggressiveness of extensive-disease SCLC generally suggests a low risk for competing causes. Conclusively, the use of CPI in patients with poor performance status appeared to be feasible and safe, but was only effective in a small proportion of patients, underscoring the need for prospective data in specific subset of patients.

NLR was identified as a robust and readily available biomarker to predict survival in patients with R/R SCLC receiving CPI in our cohort. The NLR has been proposed as a simple marker for general immune response to various stress stimuli and prognostic utility has been evaluated in the context of trauma^32^ and malignancy,^33,34^ including lung cancer treated with CPI.^35–38^ We corroborate existing data that NLR may be a valuable biomarker of prognosis in patients with SCLC. However, since the ratio has shown prognostic significance independent of the therapy used, we are cautious about interpreting it as a predictive biomarker for response to CPI therapy, especially because the response to CPI therapy was not affected by NLR. Other clinical characteristics, such as the presence of liver or brain metastases, platinum-resistant tumors, hyponatremia, and LDH above the median, have, in our view, rather prognostic significance as they indicate an advanced stage of the disease and more aggressive biology. Nevertheless, a favorable response to CPI is still possible and should not lead to the categorical exclusion of such a therapeutic option.

Our study faces some limitations, most of which are due to its retrospective nature. In particular, the heterogeneity of patients and treatment regimens as well as missing variables constrains the validity of our findings for smaller subgroups and multivariate analysis. In addition, adverse events were reported on the discretion of the treating physicians and do not meet the requirements of completeness according to Common Terminology Criteria for Adverse Events (CTCAE) standards. Yet, we believe that clinically relevant endpoints, such as permanent withdrawal from treatment and OS, allow for a reasonable estimation of the efficacy and safety of CPI in a real-world population of patients with R/R SCLC.

Given the fact that 12- and 24-month survival rates were 31.8% and 12.7%, respectively, we believe that there is a small subset of patients with a long-lasting benefit from CPI treatment as seen in other malignant diseases. This is further underscored by first-line clinical trials that have evaluated CPI-chemotherapy combinations in SCLC, in particular CASPIAN^39^ and IMPower133.^40,41^ CASPIAN and IMPower133 provided robust evidence for improved survival, and updated OS analyses revealed an 18-month survival of 34.0%^41^ (IMPower133) and 24-month survival of 23.4%^42^ (CASPIAN), respectively. Of note, other clinical trials with similar design have failed to show improved survival upon chemo-immune combinations, thus implying that not all subgroups experience equal benefit from these combination strategies. It is therefore of utmost importance to further define (clinical and molecular) subgroups that benefit most from CPI in SCLC. To this end, prospective trials like ‘BIOLUMA’ (NCT03083691) will help to define the role of CPI treatment in this aggressive malignancy.

In conclusion, CPI is of limited value in an undifferentiated R/R SCLC patient cohort, and we were not able to identify robust predictive biomarkers for therapy response and favorable survival. Clinical characteristics allow for a more fine-grained subgroup selection. Patients with good performance status, platinum-sensitive tumors, absence from liver and brain metastases, low LDH, and in particular low NLR may benefit most from CPI treatment in R/R SCLC and may facilitate long-term survival, especially when treated with CPI combination strategies. Further evaluation of these considerable patient subgroups and new combination strategies are needed to overcome the negative prognostic impact of R/R SCLC.

## Supplemental Material

sj-docx-1-tam-10.1177_17588359221097191 – Supplemental material for Clinical predictors of survival in patients with relapsed/refractory small-cell lung cancer treated with checkpoint inhibitors: a German multicentric real-world analysis Click here for additional data file. Supplemental material, sj-docx-1-tam-10.1177_17588359221097191 for Clinical predictors of survival in patients with relapsed/refractory small-cell lung cancer treated with checkpoint inhibitors: a German multicentric real-world analysis by Jan A. Stratmann, Radha Timalsina, Akin Atmaca, Vivian Rosery, Nikolaj Frost, Jürgen Alt, Cornelius F. Waller, Niels Reinmuth, Gernot Rohde, Felix C. Saalfeld, Aaron Becker von Rose, Fabian Acker, Lukas Aspacher, Miriam Möller and Martin Sebastian in Therapeutic Advances in Medical Oncology
