# Supplementary material for: Clinical predictors of survival in patients with relapsed/refractory small-cell lung cancer treated with checkpoint inhibitors: a German multicentric real-world analysis
Source: Ther Adv Med Oncol. 2022 Jun 4;14:17588359221097191. doi: 10.1177/17588359221097191 (PMC9168937; doi:10.1177/17588359221097191)
Supplement: sj-docx-1-tam-10.1177_17588359221097191 – Supplemental material for Clinical predictors of survival in patients with relapsed/refractory small-cell lung cancer treated with checkpoint inhibitors: a German multicentric real-world analysis [file sj-docx-1-tam-10.1177_17588359221097191.docx]

# Clinical predictors of poor survival in patients with relapsed / refractory small-cell lung cancer treated with checkpoint inhibitors: a German multicentric real-world analysis

Jan A Stratmann (1), Radha Timalsina (1), Akin Atmaca (2), Vivian Rosery (3), Nikolaj Frost (4), Jürgen Alt (5), Cornelius F Waller (6), Niels Reinmuth (7), Gernot Rohde (8), Felix C Saalfeld (9), Aaron Becker von Rose (10), Fabian Acker (1), Lukas Aspacher (1), Miriam Möller (11)*, Martin Sebastian (1)*

1 Department of Internal Medicine, Hematology/Oncology, Goethe University, Frankfurt, Frankfurt am Main, Germany

2 Department of Oncology and Hematology, Krankenhaus Nordwest, UCT-University Cancer Center, Frankfurt, Germany

3 Department of Medical Oncology, West German Cancer Center, University Medicine Essen, Essen, Germany

4 Charité – Universitätsmedizin Berlin, corporate member of Freie Universität Berlin, Humboldt-Universität zu Berlin, and Berlin Institute of Health, Department of Infectious Diseases and Pulmonary Medicine, Berlin, Germany

5 Department of Internal Medicine III (Hematology, Oncology, Pneumology), University Medical Center Mainz, Mainz, Germany

6 Internal Medicine I, Haematology, Oncology and Stem Cell Transplantation, Freiburg University Medical Center and Faculty of Medicine, Freiburg, Germany

7 Department of Oncology, Asklepios Clinic München-Gauting, Gauting, Germany

8 Department of Respiratory Medicine, Medical Clinic 1, University Hospital, Frankfurt, Germany

9 Department for Internal Medicine I, University Hospital Carl Gustav Carus Dresden, TU Dresden, Germany

10 Department of Internal Medicine III, Klinikum rechts der Isar, Technical University Munich, Munich, Germany

11 Department of Oncology, Martha - Maria Hospital Halle, Halle, Germany

*Authors contributed equally to the manuscript

Corresponding author: Jan Stratmann, Department of Medicine, Hematology/Oncology, Goethe University, Frankfurt, Theodor Stern Kai 7, 60596 Frankfurt am Main, Germany, Email: [jan.stratmann@kgu.de](mailto:jan.stratmann@kgu.de); Phone: 00496963015051; Fax: 004969630183655; ORCID ID: 0000-0001-7726-6622

| **Table S3** |  |  |  |  |  |  |  |  |
| --- | --- | --- | --- | --- | --- | --- | --- | --- |
| Immune-related adverse events of all grades stratified by ECOG status | | | | | | | | |
|  |  | **all patients, n=63** | | **ECOG 0 / 1, n=36** | | **ECOG 2+, n=20** | | **p value** |
| irAE: skin toxicity |  | 20 | 18.0% | 14 | 20.0% | 4 | 13.8% | 0.15 |
| irAE: gastrointestinal toxicity | | 15 | 13.5% | 8 | 11.4% | 6 | 20.7% | 0.52 |
| irAE: liver / pancreas toxicity |  | 7 | 6.3% | 2 | 2.9% | 3 | 10.3% | 0.24 |
| irAE: endocrine toxicity |  | 14 | 12.6% | 10 | 14.3% | 3 | 10.3% | 0.28 |
| irAE: lung toxicity |  | 23 | 20.7% | 13 | 18.6% | 8 | 27.6% | 0.77 |
| irAE: neurological toxicity | | 6 | 5.4% | 3 | 4.3% | 3 | 10.3% | 0.44 |
| irAE: other |  | 17 | 15.3% | 11 | 15.7% | 5 | 17.2% | 0.66 |
| irAE, immune-related adverse events; ECOG, eastern cooperative oncology group | | | | | | | | |
